# Use of artificial structures to enhance fish diversity in the Youjiang River, a dammed river of the Pearl River in China

**DOI:** 10.1002/ece3.6949

**Published:** 2020-11-11

**Authors:** Dingli Guo, Lei Zhou, Gongpei Wang, Han Lai, Sheng Bi, Xiaoli Chen, Xiaopin Zhao, Shuang Liu, Yong Luo, Guifeng Li

**Affiliations:** ^1^ Guangdong Key Laboratory of Improved Variety of Aquatic Economic Animals School of Life Sciences Sun Yat‐sen University Guangzhou China; ^2^ Southern Laboratory of Ocean Science and Engineering (Guangdong, Zhuhai) Zhuhai China; ^3^ Institute of Aquatic Economic Animals School of Life Sciences Sun Yat‐sen University Guangzhou China; ^4^ Guangdong Provincial Engineering Technology Research Center for Healthy Breeding of Important Economic Fish Guangzhou China; ^5^ College of Marine Sciences South China Agricultural University Guangzhou China; ^6^ State Key Laboratory of Ophthalmology Zhongshan Ophthalmic Center Sun Yat‐sen University Guangzhou China; ^7^ Fishery, Animal Husbandry and Veterinary Bureau of Tianyang County Baise China

**Keywords:** artificial habitat, fish abundance, fish diversity, fishery resources, species richness, the Pearl River

## Abstract

The fragmentation and homogenization of habitats have seriously affected the fishery resources of the Pearl River. To protect the fishery resources, a novel artificial habitat, constructed using bamboo and palm slices, was deployed in the Youjiang River, a tributary of the Pearl River in China. The results of field and laboratory experiments showed that fish abundance, species richness and Shannon–Wiener diversity index were higher in the artificial habitats than at the control sites. There was no significant impact on fish biomass, as the artificial habitats attracted more Cultrinae and Gobioninae fish that are of a smaller size. Artificial habitats can serve as spawning grounds for fish that produce sticky eggs and refuges that improve the survival rates of juvenile fishes. This study revealed that this novel artificial habitat created suitable habitats and suitable spawning substrate for fish, improved fish richness and diversity in the structureless freshwater ecosystem like the Youjiang River.

## Graphical abstract

The fragmentation and homogenization of habitats have seriously affected the fishery resources of the Pearl River. To protect the fishery resources, a new type of artificial habitat, consisting of bamboo and palm slices, was deployed in the Youjiang River, a tributary of the Pearl River, China. This study revealed that the new type of artificial habitat can form new habitats for fish in the Youjiang River.

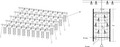

## 1 INTRODUCTION

The Pearl River is the third‐longest river in China, with an abundance fishery resources and rich fish biodiversity. The river supports 385 species of fish, with Cypriniforms being the most dominant (Zhou et al., 2018). However, aquatic habitats have been degraded dramatically, fishery resources have decreased, and fish biodiversity has been continuously threatened over the past few decades due to either direct or indirect human activities. It is well known that river impoundments and introduction of alien species may be the main anthropogenic causes of diversity loss of freshwater fishes (Allan & Flecker, 1993; Dudgeon et al., 2006; Moyle & Light, 1996; Naiman & Turner, 2000; Rahel et al., 2008). From the 1950s, more than 96,000 of dams have been constructed in the Pearl River. The dams have changed the original hydrological conditions of the river, caused river fragmentation, widened out the river, and changed the velocity of water. These have led to the loss of fish species, decreases in community diversity, and habitat destruction/defragmentation (Zeng et al., 2017). Water level fluctuations caused by dam operations are harmful to fish, due to their potential of increasing fish interactions, increase fish mortality, affect water quality, destroy littoral habitats, and expose fish eggs to the air (Yamamoto et al. 2006). Additionally, the Pearl River Basin has been invaded by alien fish species, such as Tilapia, a typical alien species introduced in China for industrialized aquaculture in the 1950s that has become firmly established in the Pearl River (Tan et al., 2012). Tilapia may feed on the eggs and larvae of other fish (Russell et al., 2012). When tilapia enter natural waters, they compete for food and habitat with native fish species, leading to the reducing or even dying out of native fish populations. In the four tributaries (Guijiang, Youjiang, Yujiang, and Zuojiang) of the Pearl River, a total of 115 species belonging to eight orders were collected from 2013 to 2015, which was a dramatically lower number than the 166 species belonging to nine orders recorded during the 1980s (Zeng et al., 2017). The abundance of native fish was lower in the fish harvest, while tilapias were found to be the dominant species. Tilapia accounted for 12.32% of the total harvest in the Youjiang River, 13.23% of the Yujiang River, and 14.06% of the Zuojiang River (Zeng et al., 2017).

Artificial habitats are constructed to mimic some of the characteristics of a natural habitat in aquatic environments and to increase structural complexity for aquatic organisms in systems where natural habitats are unavailable or absent (Bolding et al., 2004). Many types of artificial habitats have been designed and used as conservation and management tools in freshwater and marine environments, including tree branches, polypropylene ribbons, tyres, ceramic, concrete, and PVC (Santos et al., 2008; Santos, Agostinho, et al., 2011; Santos, García‐Berthou, et al., 2011; Yamamoto et al., 2014; Freitas & Petrere, 2001; Čech et al., 2012; Nash et al., 1999). Numerous studies have been conducted to elucidate the role of artificial habitats for fisheries management all over the world, including the use of artificial habitats to attract fish or other organisms and increase their abundance (Hellyer et al., 2011; Jones & Tonn, 2004; Sherman et al., 2002; Sosa‐Cordero et al., 1998; Wills et al., 2004), to provide spawning substrates and increase fish recruitment (Pickering & Whitmarsh, 1997; Sandström & Karås, 2002), to offer shelter for juvenile fish (Höjesjö et al., 2015), to mediate the effects of introduced species on native species (Rahel et al., 2008; Santos, Agostinho, et al., 2011; Santos, García‐Berthou, et al., 2011; Santos et al., 2008), and to mitigate the drawdown impacts on fishes (Benoit & Legault, 2002; Santos et al., 2008).

The primary objectives of this study are to evaluate the effects of artificial habitats on fish diversity, fish composition, and the asylum effect on juvenile fish. Fish assemblage surveys were conducted from February 2016 to November 2017 at the Donghong Village on the banks of the Youjiang River (a tributary of the Pearl River, China).

## 2 MATERIALS AND METHODS

### 2.1 Study area

**FIGURE 1:**
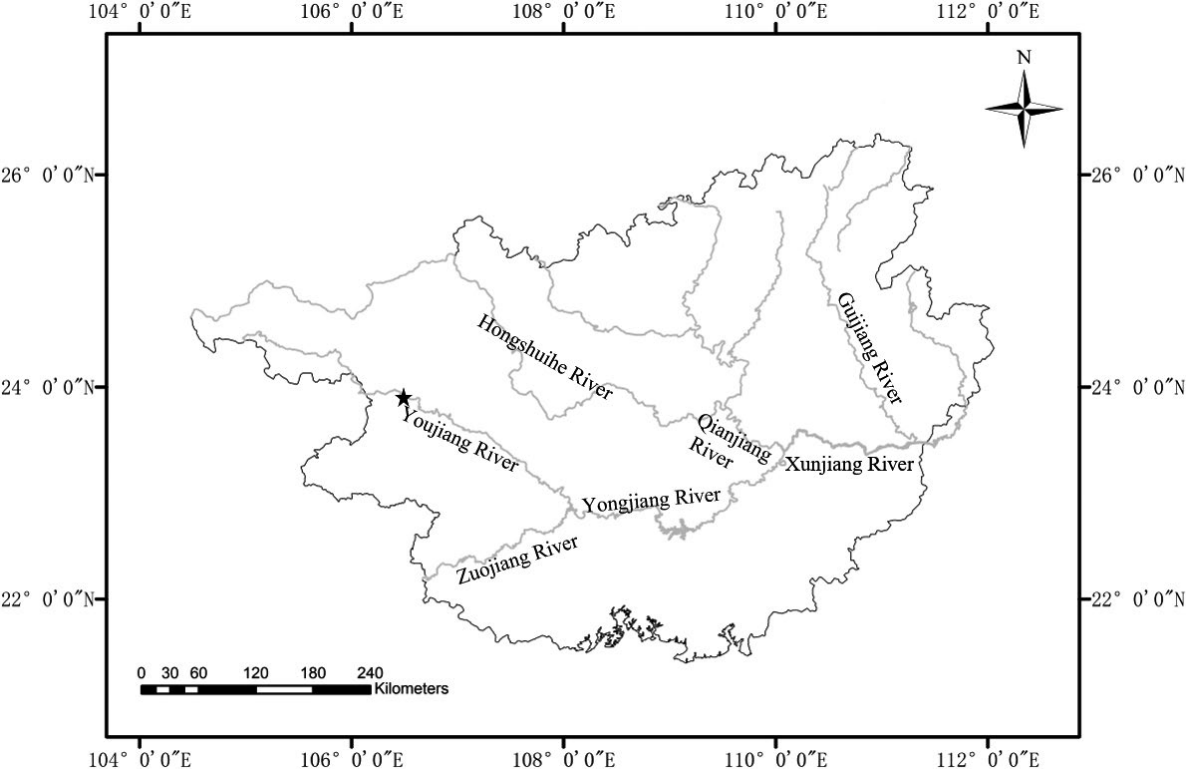
Map of the study area in the Youjiang River. The black star symbol represents the study aera (Donghong Village), gray lines represents rivers, and black lines represents geographic boundaries

The Youjiang River (a major tributary of the Pearl River) extends for 727 km and drains an area of 4.02 × 10^4^ km^2^. The Youjiang River is a major tributary of the Xijiang River, which begins in eastern Yunnan and joins the Zuojiang River near Nanning to form the Youjiang River (Zhou et al., 2018). The Youjiang River is regulated by six cascading medium/large sized dams in Wacun, Baise, Dongsun, Naji, Yuliang, and Jinjitan. In a research study done on the fish diversity of the Youjiang River, 80 fish species being recorded in a research of and the dominant species were *Hemiculter leucisculus*, *Toxabramis houdemeri*, *Oreochromis niloticus*, and *Squalidus argentatus* with relative abundances of 19.55%, 13.56%, 7.71%, and 6.55%, respectively (Zhou et al., 2018). The study area (Donghong Village) was located at 23°46.390′N 106°40.102′E, between the Baise and Naji reservoirs (Figure 1), where the width of the river was 100–150 m. The average water depth in the study area was 6 m, with a water level fluctuation of about 0.5–1 m daily due to the operation of the reservoir. The water level began to rise at about 6 p.m. and fell at 10 a.m. the next day.

### 2.2 Artificial habitat construction

**FIGURE 2:**
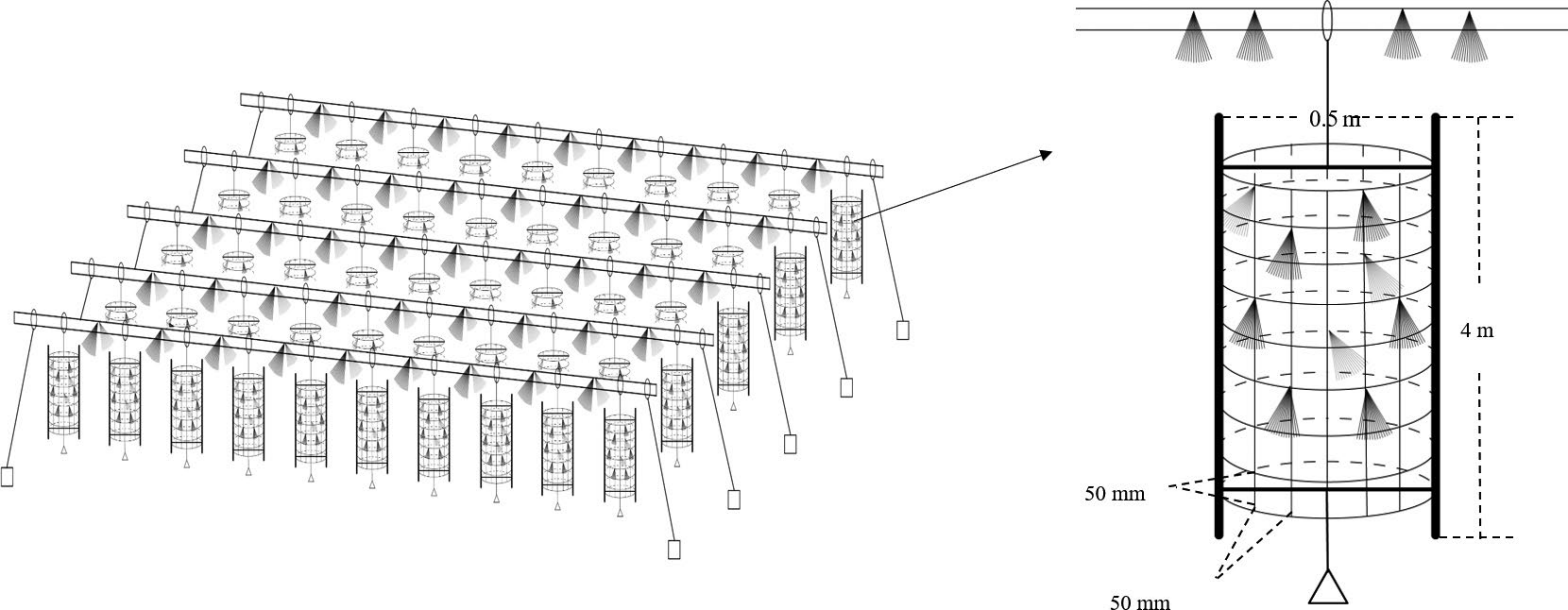
The schematic diagram of artificial habitat

The structure of the artificial habitat was divided into two parts (Figure 2): a bamboo raft on the surface of the water and a columnar frame (height: about 4 m; diameter: about 0.5 m) below the surface. The bamboo raft was made up of two bamboos (diameter: about 150 mm) laid side by side to provide buoyancy for artificial habitats and was anchored with bricks to fix them in the specific area of water. The columnar frame was a structure made of bamboo and meshes with a pore size of 50 × 50 mm. The columnar frame was reinforced by two symmetrically placed small bamboos inside the frame. The top of the column frame was suspended from the bamboo raft, while a brick was suspended from the bottom so that it stands vertically in the water. The palm slices were laid on bamboo rafts and columnar frames. Each artificial habitat consisted of a larger bamboo raft (length: 70 m) with 20 frames suspended at equal intervals, and the larger bamboo raft was made up of a series of bamboo rows.

### 2.3 Sampling design

Five sites were selected for the deployment of the artificial habitats and five for matching the control. The artificial habitats were deployed in December 2015. All the sites were otherwise structureless and were composed mainly of a clay and sand substrate. To minimize mutual interference, adjacent sites were designed to be at least 150 m apart. Fish sampling was conducted every 3 months from February 2016 (3 months after deployment of structures) to November 2017. All fish were sampled using multimesh gillnets that were 18 m long, 1.5 m height with mesh sizes between 6.25 and 60 mm of the following order: 45, 20, 6.25, 10, 55, 40, 12.5, 25, 15, 60, 35, and 30 mm (Zhou et al., 2018). Three groups of artificial habitats and three control sites were selected for sampling, and three multimesh gillnets were placed in each site. The gillnets were set nearby each artificial habitat and control site between 6:30 p.m. and 7:30 p.m. and were hauled out between 6:30 p.m. and 7:30 a.m. the following day (Zhou et al., 2018). All fish caught in each multimesh gillnet were identified at species level, measured (total length, cm), and weighed (g), separately. Then, the fish were returned to the water while still alive. The data obtained from each network were used as a sample for subsequent data analysis. The study protocols concerning the handling of the fish adhered to the requirements of the Institutional Animal Care and Use Committee of Sun Yat‐sen University, P. R. China.

To assess the viability and effectiveness of the artificial habitats as spawning substrate for fish, the palm slices were placed on the artificial habitats at different depths (0.5, 1.5, 2.5, 3.5, and 4.5 m), and during the reproduction period of *Cyprinus carpio*, the palm slices were collected from the different depths of water and the eggs clinging to the palm slices were counted.

### 2.4 Laboratory trial

Laboratory trials were carried out at the local field workstation. Laboratory‐scale artificial habitats (used as artificial refuges) used were columnar frames (height: 200 mm; diameter: 150 mm), with mesh of a pore size of 50 × 50 mm. The palm slices were laid on the artificial habitats. In the laboratory trials, the artificial habitats were hung on wooden sticks, which were placed on both sides of the tank. The artificial habitats were fixed at a depth of 100 mm underwater. The size of the tank was 600 mm (height) × 800 mm (length) × 500 mm (width). The water used in the laboratory trials was taken from the river. The average water temperature was 26.5°C, and the water depth was 350 mm, while the photoperiod was set at 14‐hr light and 10‐hr dark to simulate summer conditions. An oxygenator was used in each tank for aeration.

The juvenile tilapia and *Squalidus argentatus* were captured from the study area using a cage net, and total length (TL) was measured to the nearest mm (12.2 ± 0.39 mm and 17.1 ± 1.4 mm, respectively, mean ± *SE*). The conditions simulated were as follows: (a) 30 juvenile tilapias × a predator (*Clarias gariepinus*) with an artificial refuge both present and absent, (b) 30 juvenile *S. argentatus* × a predator with an artificial refuge both present and absent, and (c) 15 juvenile tilapia, 15 juvenile *S. argentatus* × a predator with an artificial refuge both present and absent. Each treatment replicated three times.

In these trials, juvenile tilapias, *S. argentatus,* and the predator were released into each tank and allowed to acclimatize to laboratory conditions for 30 min, and the juvenile fish and the predator were separated using a baffle placed at the center of the tank. The number of juvenile fish that survived was recorded at 1, 2, 4, 12, 24, and 48 hr. Mean *C. gariepinus* size (total length) were kept constant for each trial (1,489.6 ± 35.8 mm).

### 2.5 Data analysis

Generalized estimating equations (GEEs) in SPSS 21.0 (SPSS Inc.) were used to test the impact of habitat type, sampling time, and their impact on abundance, biomass, fish size, richness (species number), and Shannon–Wiener diversity index. GEEs are an extension of the generalized linear model (GLM) in that they allow for the adjusting of correlations between observations (Ziegler & Vens, 2010). GEEs were used as available in the SPSS 22 software package, by applying a normal distribution and identity link functions for all analyses. Then, pairwise comparisons were performed to test for differences between artificial structures and control sites per month. When *p* < 0.05, a statistically significant difference is considered.

## 3 RESULTS

### 3.1 Fish assemblages and composition

In total, 3,276 individual fish of 35 fish species, representing four orders and nine families, were recorded in the natural and artificial habitats. Overall, 2,251 individual fish of 33 species (besides *Pseudorasvora parva* and *Clonorchis sinensis*) were found at artificial habitats, while 1,025 individual fish of 29 species (besides *Clarias gariepinus, Aristichthys nobilis, Pelteobagrus fulvidraco, Ancherythroculter lini, Schistura fasciolata, Odontobutis sinensis*) were caught at the control sites. In terms of fish abundance, two species of tilapia (*Oreochromis mossambicus and Oreochromis niloticus*) and four species of cyprinidae (*Toxabramis houdemeri, Hemiculter leucisculus, Rhodeus ocellatus, and Huigobio chinssuensis*) were dominant at both the artificial habitats and control sites.

During the formation of fish communities, the artificial habitats were primarily colonized by omnivorous fishes, such as *T. houdemeri*, *H. leucisculus, Squalidus argentatus, and* tilapia (*O. mossambicus and O. niloticus*), and these species settled in artificial habitats throughout the study period. Subsequently, other omnivorous species, such as *H. chinssuensis, Squalidus wolterstorffi, R. ocellatus, and Rhinogobius giurinus,* migrated into artificial habitats. Eighteen months later, benthic fishes, such as *Mastacembelus armatus, P. fulvidraco, Pelteobagrus vachelli, C.gariepinus and O. sinensis,* were recorded at artificial habitats. Additionally, during the breeding season, *Cyprinus carpio, Carassius auratus and Osteocheilus salsburyi* appeared at artificial habitats.

**FIGURE 3:**
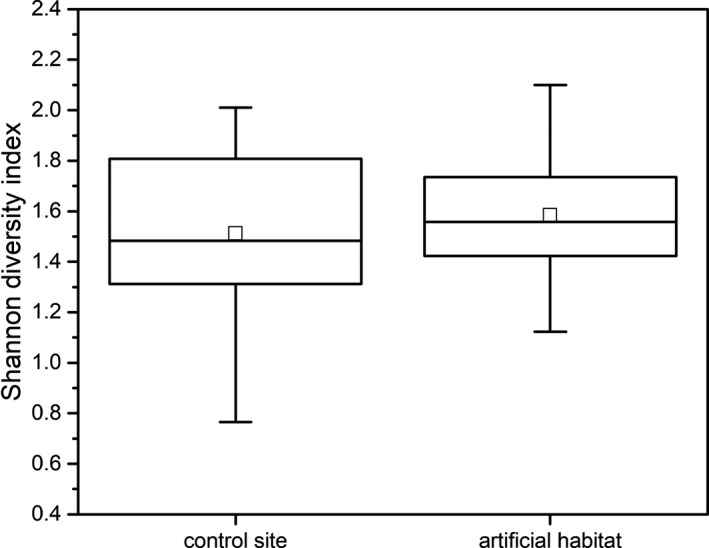
Box plot of fish Shannon diversity index at artificial habitats and control sites

Fish richness (number of species) (GEE: *Wald chi‐square* = 17.962, *df* = 1, *p* < 0.01), fish abundance (GEE: *Wald chi‐square* = 35.479, *df* = 1, *p* < 0.01), and biomass (GEE: *Wald chi‐square* = 5.431, *df* = 1, *p* = 0.02) varied significantly between artificial habitat and control site, but not for diversity (GEE: *Wald Chi‐Square* = 0.827, *df* = 1, *p* = 0.363) (Figure 6). Fish richness and abundance were higher in the artificial habitats than at the control sites (Table 1, Figure 3). However, a similar trend was not found for fish biomass, because cichlids were dominant at the control sites and accounted for most of the total biomass, with the average weight of the tilapias being higher than that of other fish. Fish richness (number of species) (GEE: *Wald chi‐square* = 33.715, *df* = 7, *p* < 0.01), fish abundance (GEE: *Wald chi‐square* = 26.827, *df* = 7, *p* < 0.01), biomass (GEE: *Wald chi‐square* = 20.589, *df* = 7, *p* = 0.004), and diversity (GEE: *Wald chi‐square* = 29.472, *df* = 7, *p* < 0.01) varied significantly with time. There were no significant habitat × time interaction in fish abundance (GEE: *Wald chi‐square* = 8.222, *df* = 7, *p* = 0.313), biomass (GEE: *Wald chi‐square* = 6.669, *df* = 7, *p* = 0.464), richness (GEE: *Wald chi‐square* = 5.686, *df* = 7, *p* = 0.557), and diversity (GEE: *Wald chi‐square* = 10.422, *df* = 7, *p* = 0.166) (Table 1).

**TABLE 1:** Habitat and temporal variations of community attributes (a), and species abundance (b), and size of the fishes (c)

	Time			Habitat			Time × Habitat		
	Wald Chi‐Square	*df*	*p*	Wald Chi‐Square	*df*	*p*	Wald Chi‐Square	*df*	*p*
(a) Community attributes									
Abundance	26.827	7	<0.01	35.479	1	<0.01	8.222	7	0.313
Biomass	20.589	7	0.004	5.431	1	0.02	6.669	7	0.464
Richness	33.715	7	<0.01	17.962	1	<0.01	5.686	7	0.557
Diversity	29.472	7	<0.01	0.827	1	0.363	10.422	7	0.166
(b) Abundance									
*H. leucisculus*	33.263	7	<0.01	3.650	1	0.056	4.717	7	0.694
*R. ocellatus*	18.322	7	0.011	11.75	1	0.001	11.562	7	0.116
*T. houdemeri*	13.955	7	0.052	21.793	1	<0.01	7.639	7	0.365
*H. chinssuensis*	27.769	7	<0.01	8.108	1	0.004	5.886	7	0.553
*S. argentatus*	12.394	7	0.088	0.003	1	0.955	10.462	7	0.164
Tilapia	21.887	7	0.003	1.223	1	0.269	6.754	7	0.455
(c) Size									
*H. leucisculus*	11.247	7	0.128	4.943	1	0.026	16.633	7	0.02
*R. ocellatus*	10.639	7	0.155	4.192	1	0.041	9.712	7	0.205
*T. houdemeri*	7.599	7	0.369	6.977	1	0.008	1.287	7	0.989
*H. chinssuensis*	22.651	7	0.002	1.056	1	0.304	6.014	7	0.538
*S. argentatus*	13.718	7	0.056	0.162	1	0.687	8.897	7	0.26
Tilapia	17.377	7	0.015	2.429	1	0.119	4.174	7	0.76

Note

*F* values, degrees of freedom, and significance of generalized estimating equations (GEEs) are shown.

**FIGURE 4:**
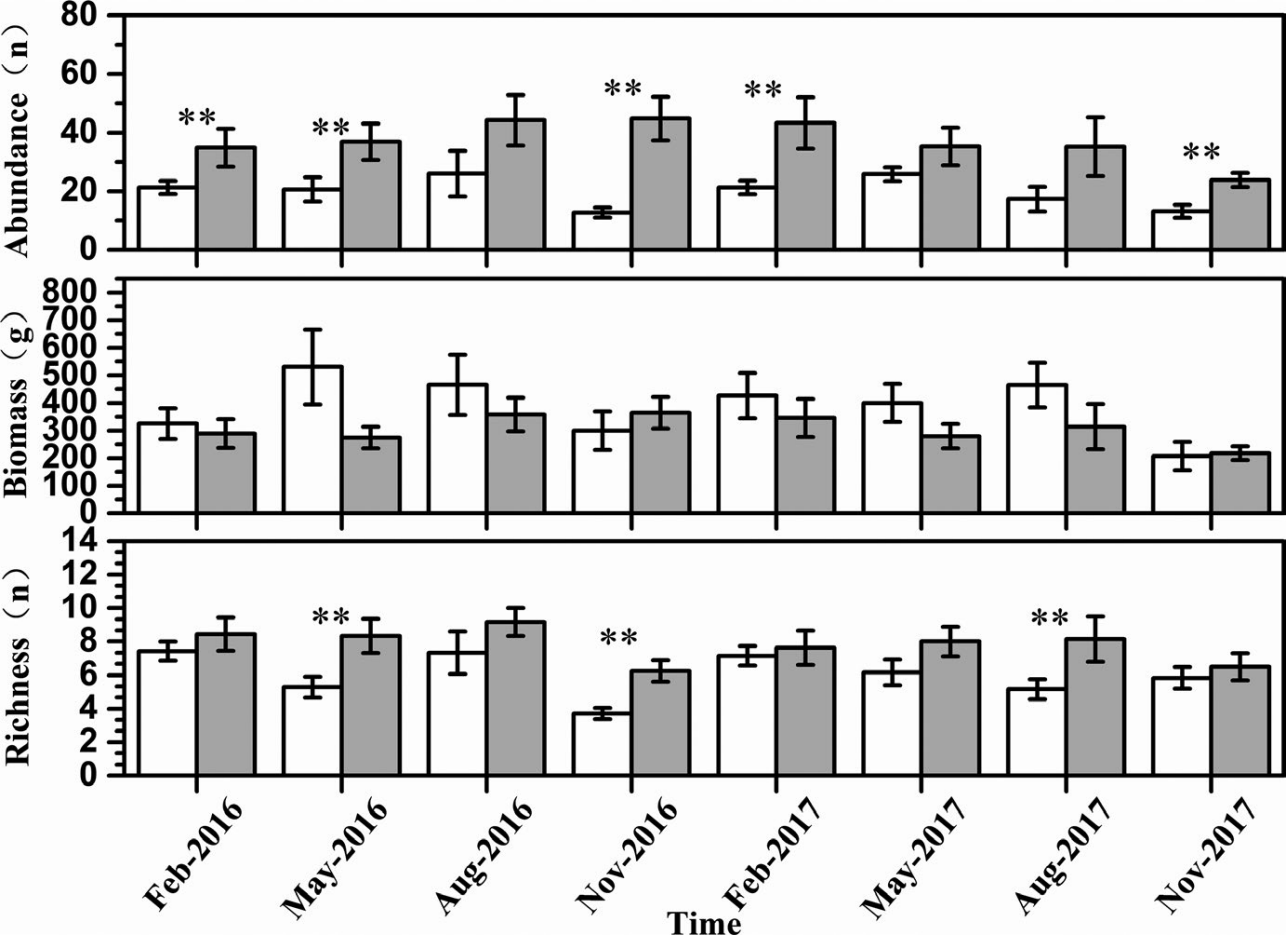
Mean species richness, abundance, biomass at the artificial habitats (gray bars), and the control sites (white bars). Data were expressed as mean ± *SD*, **significant between artificial habitats and control sites at the 0.05 level

**FIGURE 5:**
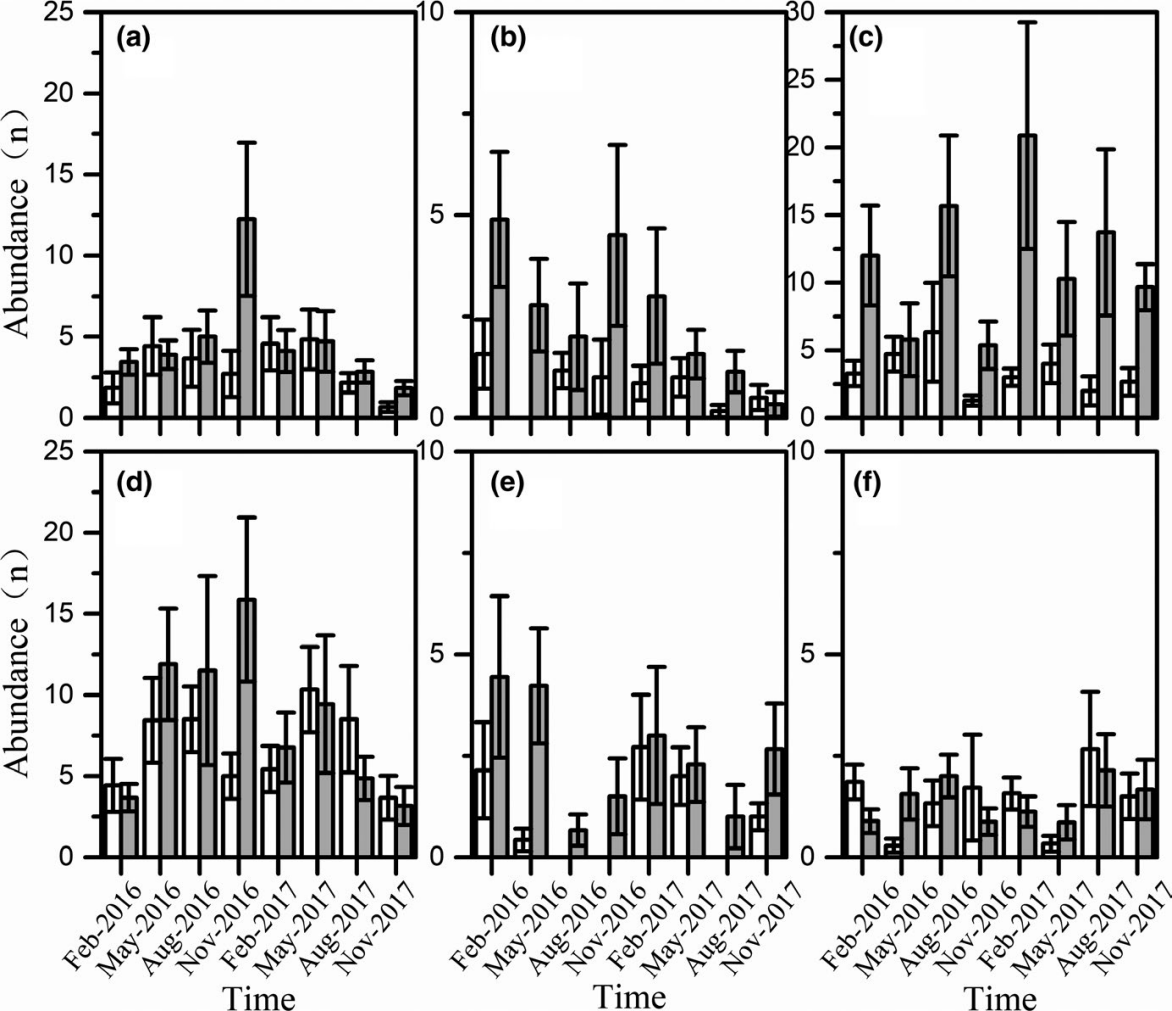
Mean abundance of the dominant species at the artificial habitats (gray bars) and the control sites (white bars). Data were expressed as mean ± *SD*. (a‐*H. leucisculus*, b‐*R. ocellatus*, c‐*T. houdemeri*, d‐tilapia, e‐*H. chinssuensis*, f‐*S. argentatus*)

For the dominant species (Table 1, Figure 4, Figure 5), the abundance and size of the dominant species also showed significant variation among habitat and time. The abundance of *H. leucisculus* (GEE: *Wald chi‐square* = 33.263, *df* = 7, *p* < 0.01)*, R. ocellatus* (GEE: *Wald chi‐square* = 18.322, *df* = 7, *p* = 0.011)*, H. chinssuensis* (GEE: *Wald chi‐square* = 27.769, *df* = 7, *p* = 0.166), and tilapia (GEE: *Wald chi‐square* = 21.887, *df* = 7, *p* = 0.003) varied significantly with time. *R. ocellatus* (GEE: *Wald chi‐square* = 11.75, *df* = 1, *p* = 0.001)*, T. houdemeri* (GEE: *Wald chi‐square* = 21.793, *df* = 1, *p* < 0.01), and *H. chinssuensiss* (GEE: *Wald chi‐square* = 8.108, *df* = 1, *p* = 0.004) were significantly more abundant in artificial habitats than at control sites.

**FIGURE 6:**
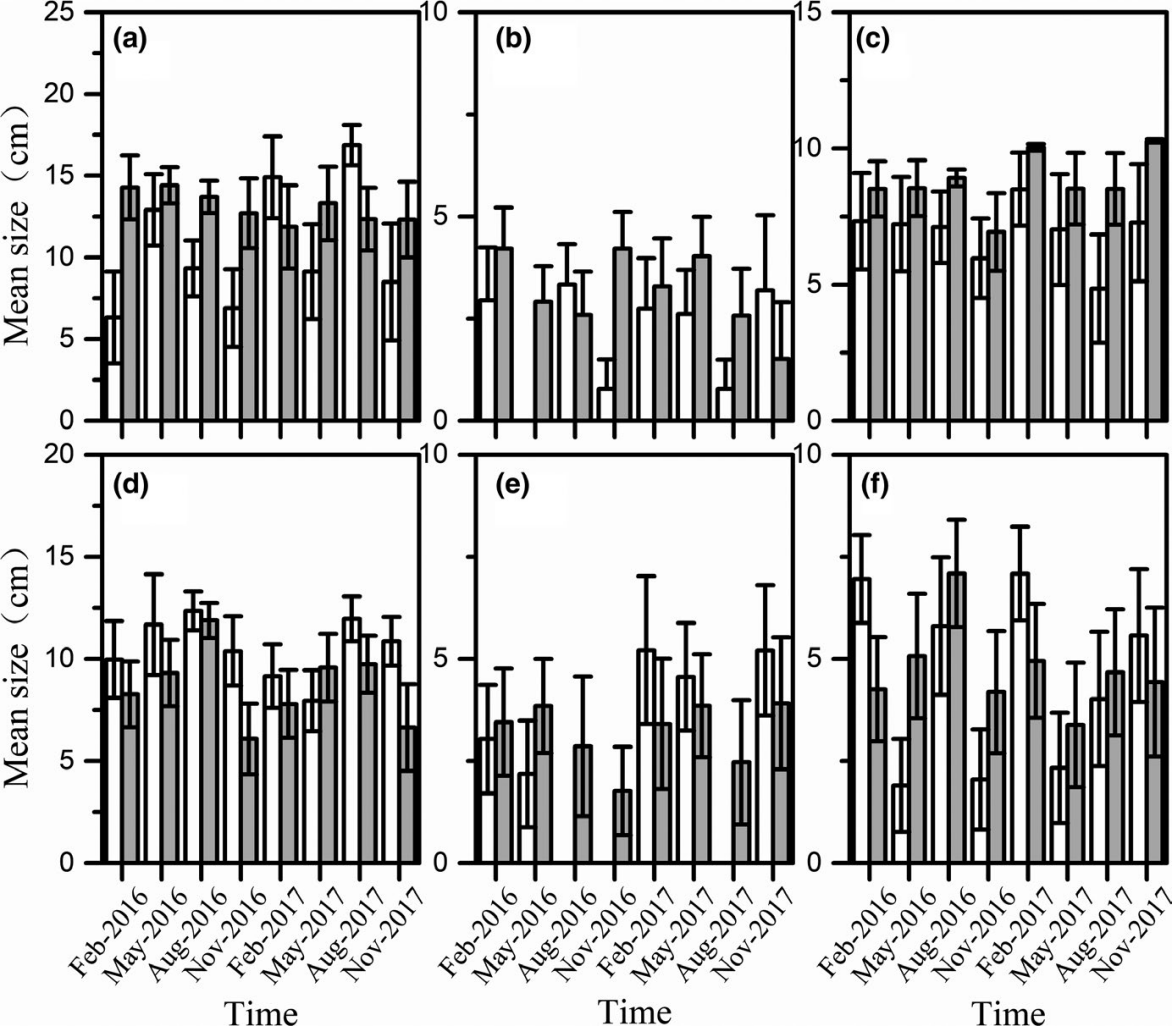
Mean size of the dominant species at the artificial habitats (gray bars) and the control sites (white bars). Data were expressed as mean ± *SD*. (a‐*H. leucisculus*, b‐*R. ocellatus*, c‐*T. houdemeri*, d‐tilapia, e‐*H. chinssuensis*, f‐*S. argentatus*)

The abundance of *H. leucisculus, S. argentatus, and* tilapia was not significant between habitats. However, it is worth to note that the abundance of tilapia was higher in artificial habitats than at the control sites until February 2017, but thereafter, the abundance of tilapia was higher at control sites than in the artificial habitats. The abundance of the six dominant species was not significant for the habitat × time interaction. The size of *H. leucisculus* (GEE: *Wald chi‐square* = 4.943, *df* = 1, *p* = 0.026)*, R. ocellatus* (GEE: *Wald chi‐square* = 4.192, *df* = 1, *p* = 0.041), and *T. houdemeri* (GEE: *Wald chi‐square* = 6.997, *df* = 1, *p* = 0.008) varied significantly between habitats, while the three other species showed no significant variation. The size of *H. chinssuensis* (GEE: *Wald chi‐square* = 22.651, *df* = 7, *p* = 0.002) and tilapia (GEE: *Wald chi‐square* = 17.377, *df* = 7, *p* = 0.015) changed significantly along with time. Only the size of *H. leucisculus* (GEE: *Wald chi‐square* = 16.633, *df* = 7, *p* = 0.02) showed a habitat × time interaction.

### 3.2 Impacts on the survival of juvenile fish

**FIGURE 7:**
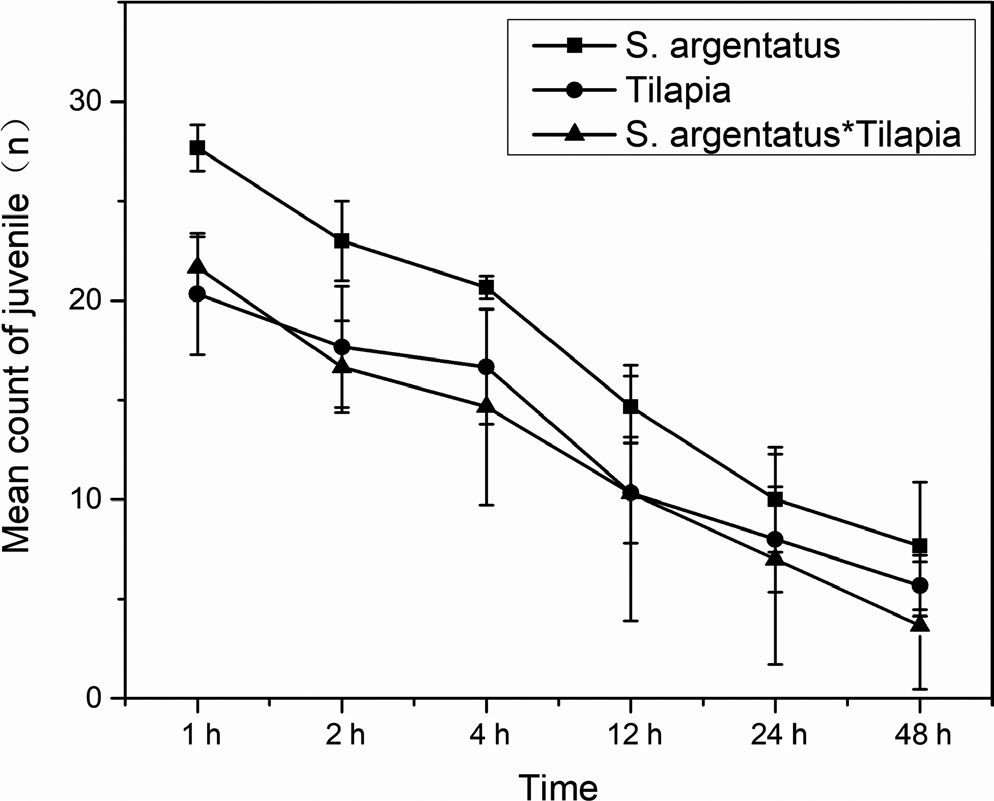
Mean number of surviving juvenile fish in treatments with artificial habitats and the predator (*C. gariepinus*)

In the treatment groups without the artificial habitat, all the juvenile (tilapia or *S. argentatus*) were preyed by *C. gariepinus* within 2 hr. When the artificial habitat was present, the survival rates of the juvenile tilapia and *S. argentatus* greatly improved. The *S. argentatus* with *C. gariepinus* was on average 25.56%, while the 48‐hr survival rate for tilapia was on average 18.89% (Figure 7). In treatments without the artificial habitat, juvenile tilapia, and *S. argentatus* together with *C. gariepinus*, all juvenile fish were preyed upon by the *C. gariepinus* within 2 hr. However, the survival rates of the juvenile tilapia and *S. argentatus* were 8.89% and 15.56%, respectively, in the presence of the artificial habitats (Figure 7). The results of the laboratory trials indicated that the deployment of the artificial habitat could effectively protect juvenile fish from predators.

### 3.3 Artificial habitats as spawning grounds

**FIGURE 8:**
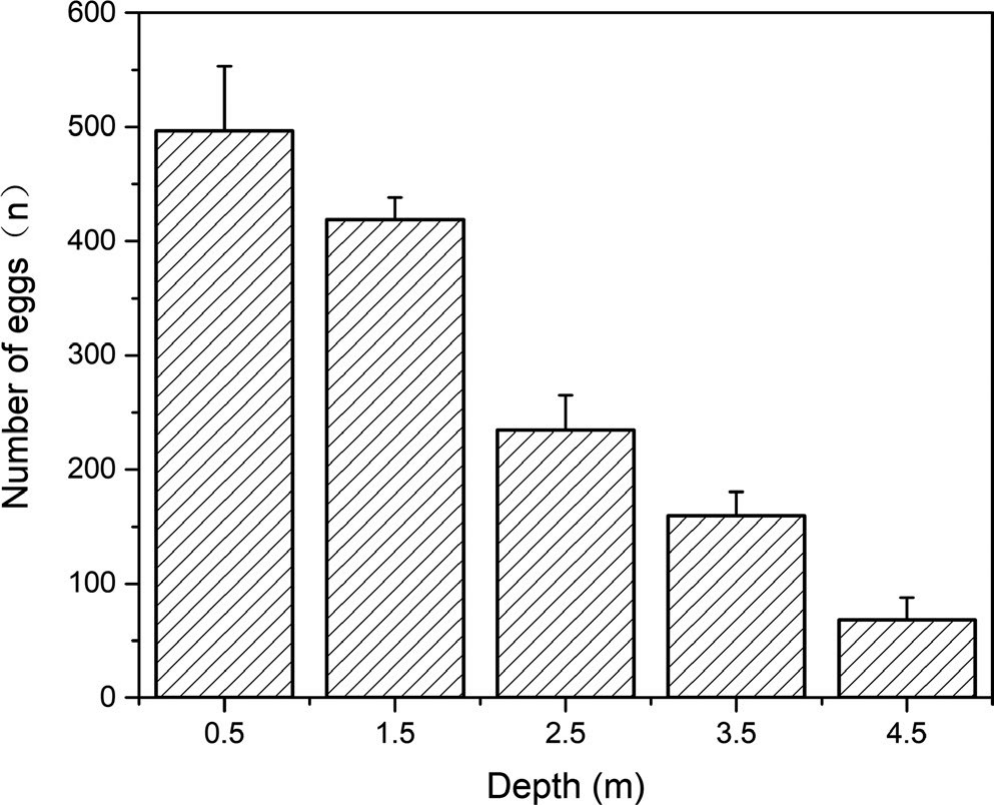
Mean number of eggs per palm slice at different water depth

The palm slices placed in the artificial habitats was designed to act as a substrate for fish spawning. In March 2107, during the reproduction period of *C. carpio*, the palm slices were collected from different depths of water and the number of eggs clinging to each palm slice was counted. Fish eggs were identified as *C. carpio* after hatching. The number of eggs on each palm slice was 46–571 individuals, and the number decreased as water depth increased. There were no aquatic plants to serve as spawning grounds for fish at the control sites, and no eggs were collected. The mean number of eggs on each palm slices at different depths of water demonstrated that palm slices placed between 0.5 m and 1.5 m were most useful for fish to lay their eggs (Figure 8).

## 4 DISCUSSION

### 4.1 Influence of artificial habitats on fish diversity

Artificial habitats can function as fish attractors, improving fish species richness and enhancing fish biomass (Powers et al., 2003). The results in this study showed that fish preferentially selected the artificial habitats over the natural habitats, as revealed by differences in fish species richness and abundance. The species richness, the number of total abundance, and diversity were higher in the artificial habitats than that at control sites. Other research studies carried out at other areas (Freitas & Petrere, 2001; Freitas et al., 2002, 2005; Gois et al., 2012; Huang et al., 2017; Santos, Agostinho, et al., 2011; Santos, García‐Berthou, et al., 2011; Santos et al., 2008) have reported similar results. For example, Santos, Agostinho, et al. (2011) and Santos, García‐Berthou, et al. (2011) used large macrophytes as artificial habitats in a Mediterranean reservoir, and the results showed that the abundances of *Perca fluviatilis*, *Rutilus rutilus,* and *Abramis brama* in the artificial habitats were significantly higher than that of the control sites, and artificial habitats were able to alleviate the negative impacts of reservoir water level fluctuations on fish communities.

The fish in the artificial habitats were affected by the availability of habitat, the composition of the fish assemblage, and their abundance (Bolding et al., 2004). *T. houdemeri*, *H. leucisculus, S. argentatus, and* tilapia (*O. mossambicus and O. niloticus*) were dominate at most rivers in the Pearl River Basin, and they were primarily colonized in the artificial habitats. As mentioned above, dam construction had caused serious problems of simplifying and homogenizing the habitat structure of the Pearl River Basin. In our study, there were no macrophytes in the study areas, so the higher level of heterogeneity found at the artificial habitats could be a key factor that may explain the comparatively richer and more balanced fish assemblages observed, as habitats with increased complexity may induce the formation and maintenance of richer communities (Freitas & Petrere, 2001; Meerhoff et al., 2007). In this study, the artificial habitats were designed to be more physically complex than the natural habitats (structureless with no hydrophytes), since the palm slices that were hung on the frames acted as artificial submerged plants that provided extra habitats for the fish. The cylindrical frameworks of the artificial habitats were deployed vertically into the water, forming a semi‐enclosed space in the mid‐water, which afforded a more physically complex space for fish, and was more effective in holding fish. In addition, the periphyton that had grown on the frameworks and palm slices provided more food, compared with control sites from which hydrophytes were absent (Santos et al., 2008). This was also an important factor for the accumulation of fish in the artificial habitats.

Artificial habitats can alleviate competition between alien species and indigenous fish and contribute to the coexistence of different fish species (Sandström & Karås, 2002; Westhoff et al., 2013). Currently, tilapia has been widely distributed in the Pearl River (Tan et al., 2012). Tilapias are tolerant of wide levels of fluctuations in salinity, dissolved oxygen, temperature (Avella et al., 1993; Farmer & Beamish, 1969; Febry & Lutz, 1987; Zale & Gregory, 1989), and have high rates of fecundity (Duponchelle et al., 1998), rapid growth rates (Liti et al., 2005), and omnivorous feeding habits (ElSayed, 1999). These characteristics have made tilapia popular in aquaculture, but at the same time have allowed tilapia to successfully invade aquatic ecosystems worldwide and proliferate in these areas (Costapierce, 2003; Courtenay, 1997; Crutchfield, 1995; Faunce & Paperno, 1999; Peterson et al., 2004, 2006), and have altered the native community habitat, even to the point of causing species extinctions (Martin et al., 2010; Mccrary et al., 2007; Russell et al., 2012). In this research study, tilapias accounted for 34.05% of the total catch in control sites and more than 22.61% in artificial habitats. The relative abundance of tilapias in the control sites was higher throughout the study period. In the artificial habitat, the relative abundance of tilapias was higher in the early and middle stage of the study, but decreased in the late stage. This may be because the artificial habitats were able to alleviate competition for food and habitat between the tilapias and species of Cyprinidae.

Yamamoto et al. (2014) revealed that artificial habitats may provide habitat for some rare species. They found that 26 species of fish, which were rare species in the area, were recorded only in artificial habitats; the artificial habitats could provide shelter for these species during drought periods and reduce their risk of predation (Yamamoto et al., 2014). In our study, *C. gariepinus*, *A. nobilis*, *P. fulvidraco*, *A. lini*, *S. fasciolata,* and *M. fukiensis,* which have relatively low abundances in the Pearl River, were recorded only in the artificial habitats. This result indicates that the novel artificial habitats used in our study could provide habitats for these rare species.

### 4.2 Artificial habitats form multifarious habitats for fish

Artificial habitats have been widely used for fishery resource conservation worldwide, but the materials, types, sizes, and deployment positions of the habitats were found to have different effects on fish communities (Bolding et al., 2004; Jaxionharm & Szedlmayer, 2015; Lindberg et al., 2006; Lingo & Szedlmayer, 2006; Santos et al., 2008). For example, Lindberg et al. (2006) found that larger reefs showed greater densities and abundance of fish. Santos et al. (2008) used polypropylene wire bundles as artificial habitats to assess the effect of artificial habitats in a new tropical reservoir in Brazil. The results revealed that the density of the habitat and its location in the water layer were key factors that affected fish utilization efficiency. Some researchers have used different materials, such as tiles and branches, as spawning substrates for fish, to make up for the lack of suitable substrates in natural habitats (Nash et al., 1999). Sandström and Karås (2002) used structures made of spruce bundles that were covered with nets as nursery habitat for young fish, and the results showed that the artificial refuges generally attracted young fish by reducing predation risk.

The study area used in this research is located between the Baise and Naji reservoirs. After the construction of the reservoir, the water level of the river frequently rises, due to the operation of the reservoir, with a water level fluctuation of about 0.5–1 m daily. Previous studies have shown that river impoundments may be one of the main anthropogenic causes of diversity loss in freshwater fishes (Agostinho et al., 1999; Beklioglu et al., 2006; Dudgeon et al., 2006). When the water level rises, the vegetation on the littoral zone of the river increases shelter and food availability and improves reproductive success, while drawdowns often heighten biotic interactions and may increase fish mortality, through impairment of water quality, destruction of littoral habitats, and exposure of eggs to desiccation (Santos et al., 2004; Sutela et al., 2002). On the other hand, the invasion of tilapias exacerbated the decline of fishery resources. As mentioned above, tilapias have become the dominant species in most tributaries of the Pearl River. Tilapias are usually omnivorous and devour eggs and juveniles of other fish species. In particular, tilapia larvae may feed on smaller fish species (Russell et al., 2012). Once tilapias have invaded natural waters, they gain an advantage in competing for food and habitats with indigenous fish, resulting in the rapid decline or even extinction of indigenous fish species (Kolar & Lodge, 2002; Martin et al., 2010; Mccrary et al., 2007; Peterson et al., 2006).

Considering the fluctuations of the water level and the effects of tilapia invasion, we designed artificial habitats that can automatically be adjusted based on water level changes using bamboo rafts laid on the surface of the river, so that the artificial habitats remain in the same relative position. The cylindrical bamboo frames attached to the bamboo rafts were vertically placed in the water. The palm slices laid on the bamboo rafts and the cylindrical frames acted as hydrophytes and a spawning substrate for fish. The bamboo cylindrical frames with meshes on the surface increased the complexity of the artificial habitats, allowing them to more effectively serve as refuges for fish by enhancing the complexity and availability of habitats.

Westhoff et al. (2013) reported that the construction of artificial habitats can improve the heterogeneity of fish habitats, and they found using laboratory experiments that artificial habitats could provide shelter for *Fundulus julisia* juveniles and improve their survival rate. In this research study, we also achieved similar results. The results of the laboratory trials indicated that the deployment of artificial habitats could protect juvenile fish from predators with the largest positive effect being on juvenile *S. argentatus*. The presence of artificial habitats delayed juvenile tilapia and *S. argentatus* mortality in the laboratory trials and improved the survival rates of the juvenile. This result also proved that artificial habitats can alleviate the effects of alien species (tilapia) on indigenous fish (Sandström & Karås, 2002; Westhoff et al., 2013).

Artificial habitats can provide spawning substrates and increase fish recruitment (Pickering & Whitmarsh, 1997; Sandström & Karås, 2002). For example, Nash et al. used tree branches as spawning substrates for local fish, making up for the lack of fish spawning matrices in the natural environment, and significantly increased the hatching rate of fish eggs (Nash et al., 1999). In this study, during the reproduction period of *C. carpio*, the palm slices were collected from different depths of water and the number of eggs on each was counted. The results showed that the number of eggs on each palm slice ranged from 46 to 571 individuals and decreased as water depth increased.

For a long time, in China, artificial habitats have been only used for fish spawning in freshwater, and these artificial habitats have mostly been deployed on the surface of the water. Additionally, the structure and the function of artificial habitats have not been able to meet the needs of fish that feed at different water layers and different life stages at the same time. The artificial habitats designed in this research study were semiclosed and spread throughout different layers of the water. This design could increase the spatial heterogeneity of the habitat and meet the needs of different life stages of fish, by increasing the spawning, foraging, and predation avoiding ability of the fish. All the above results showed that the artificial habitats were effective for use as shelters and spawning grounds.

### 4.3 Implications for conservation and management

The biodiversity of freshwater ecosystems has been threatened for a long time, such as habitat loss and degradation, caused by anthropogenic activities (Chen et al., 2012; Dudgeon et al., 2006; Fiedler & Truxa, 2012). As IPBES reports: Inland waters and freshwater ecosystems have the highest rates of decline due to land‐use change, water extraction, exploitation, pollution, climate change, and invasive species. By 2000, the size of wetlands was only 13% of what it was in 1,700, and it declined even more rapidly from 1970 to 2008 (by 0.8% per year). The establishment of dams for water storage and tilapia invasion maybe the two main reasons for the decline of fishery resources in the Youjiang River. Fluctuations in the water level have caused damage to aquatic vegetation, and habitats become unavailable to aquatic organisms (Santos, Agostinho, et al., 2011; Santos, García‐Berthou, et al., 2011), resulting in the decrease of fish biodiversity. Additionally, the invasion of tilapia has aggravated changes to fish community structure. In China, for many years stock enhancement has been used to compensate for the decline of fishery resources in freshwater. However, the fish species of stock enhancement were limited, and its proliferation did not fundamentally solve the problem of diversity decline and habitat degradation. As an effective fishery management measure, artificial habitat has been widely used all over the world (Santos, Agostinho, et al., 2011; Santos, García‐Berthou, et al., 2011; Yamamoto et al., 2014). Benefiting from the bamboo rafts, the artificial habitats used in our research were able to self‐adjust to the variation of water level as the “stereoscopic artificial floating wetlands” (Huang et al., 2017) and can be used to compensate for the destruction of habitats. On the one hand, the bamboo cylindrical frames increased the complexity of habitat just like the artificial habitats made of “large woody debris” (Yamamoto et al. 2006) and thus increased the density of fish; on the other hand, the meshes on the surface of the frame formed many small interstitial spaces played a similar role with the artificial structure made of spruce bundles with surrounding nets (Sandström & Karås, 2002), which acted as “protective devices” for young fish that increased the refuge capacity by reducing predation risk. Imitations of hydrophyte, such as brushwood bundles (Nash et al., 1999), were often used to provide spawning substrates for fish. The palm slices laid on bamboo rafts and columnar frames played the same role in our study. Our experiments demonstrate that the deployment of artificial habitats may be an effective measure for the restoration of fish habitats in the Youjiang River and other freshwater ecosystems where natural habitats have been damaged.

## CONFLICT OF INTEREST

The authors declared that they have no conflicts of interest to this work.

## AUTHOR CONTRIBUTIONS

**Dingli Guo:** Conceptualization (lead); data curation (lead); investigation (lead); methodology (lead); writing – original draft (lead); writing – review and editing (lead). **Lei Zhou:** Conceptualization (equal); investigation (equal); methodology (equal). **Gongpei Wang:** investigation (equal); methodology (equal). **Han Lai:** Investigation (equal). **Sehng Bi:** Investigation (equal). **Xiaoli Chen:** Investigation (equal). **Xiaopin Zhao:** Investigation (equal). **Shuang Liu:** Resources (equal). **Yong Luo:** Resources (equal). **Guifeng Li:** Conceptualization (equal); methodology (equal); project administration (equal); validation (equal); writing – original draft (equal); writing – review and editing (equal).

## Data Availability

The data that support the findings of this study are available on request from Dryad, Dataset, https://doi.org/10.5061/dryad.2280gb5nf.
